# Artificial intelligence as a tool for cognitive impairment screening: Patient perspectives about benefits and limitations

**DOI:** 10.1002/bsa3.70005

**Published:** 2025-03-04

**Authors:** Heather M. Wurtz, Margaret Manchester, Tatyana Valteau, Hannah Hanson, Catherine Scipion, Alex Federman, Jalayne J. Arias

**Affiliations:** 1 Department of Anthropology and Human Rights Institute, University of Connecticut, Storrs, Connecticut, USA; 2 Department of Population and Public Health Sciences, Keck School of Medicine, University of Southern California, Los Angeles, California, USA; 3 Department of Health Policy and Behavioral Sciences, School of Public Health, Georgia State University, Atlanta, Georgia, USA; 4 Division of General Internal Medicine, Icahn School of Medicine at Mt. Sinai, New York, New York, USA

**Keywords:** artificial intelligence, cognitive impairment, patient perspectives, screening

## Abstract

**Introduction:**

This paper presents findings from a qualitative study exploring the perspectives of primary care patients on the use of artificial intelligence (AI) to synthesize data from the electronic medical record (EMR) and audio recordings of patient speech as a way to detect cognitive impairment.

**Methods:**

We conducted qualitative interviews with a racially and educationally diverse sample of primary care patients (*N* = 31) from a large healthcare system in New York City, New York. Interviews focused on the use of AI to synthesize data from the EMR and audio recordings of patient speech. Data were analyzed using content analysis.

**Results:**

Participants reported that AI technology could improve diagnostic accuracy and avoid human errors in the evaluation and diagnosis of cognitive impairment, while also improving clinical efficiency for clinicians. They also expressed concerns about the use of AI tools for screening, such as risk of misdiagnosis, loss of confidentiality, and psychological distress from learning of detected cognitive impairment.

**Conclusion:**

The perceived benefits reported by participants regarding the use of AI screening tools suggest that some patients might support the provision of AI screening in clinical settings. However, participants’ concerns about the potential harms of AI screening tools underscore the need for clear and transparent communication about how results are generated and interpreted. Clinicians need training and other forms of structural support to improve their literacy in AI-driven tools and their ability to integrate adequate approaches to information sharing into clinical practice.

## 1 | INTRODUCTION

Early detection of cognitive impairment can optimize treatment approaches and support healthcare decision-making by patients and their families.^1^ However, despite Medicare coverage of cognitive impairment screening through annual wellness exams,^2^ screening rates in clinical settings remain low. Additionally, the United States Preventive Services Task Force has not endorsed routine screening for cognitive impairment, reflecting a broader uncertainty regarding the value of screening. Despite barriers for early detection, the field has experienced a shift in clinical approaches to dementia and cognitive impairment, including the emergence of disease-modifying treatments for Alzheimer’s disease. Alongside these developments, there is an increasing awareness of the need to support individuals affected by dementia-related syndromes and cognitive impairment. The evolution of clinical care for this population underscores the need for improved methods that support early and accurate diagnosis of cognitive impairment.

Artificial intelligence (AI) holds the potential to revolutionize clinical care for cognitive impairment through machine learning (ML) and associated models (eg, neural networks). AI methods, including ML and natural language processing (NLP), offer novel solutions to address existing needs for the early diagnosis of cognitive impairment and associated dementia syndromes. ML algorithms can improve with experience to make predictions or identify cases with a high level of accuracy. Studies have shown that ML methodologies can detect or predict cognitive impairment at high levels of performance, as indicated by area under the receiver operator characteristic (AUC) curve of 0.9 and higher using a variety of data sources, including electronic medical records (EMRs), speech audio recordings, and motion sensor data.^3–6^ These technologies are currently being tested in clinical practice and may soon be implemented for clinical applications,^7,8^ such as EMR-embedded algorithms for the identification of patients with mild cognitive impairment and early-stage dementia that trigger clinician-facing decision supports.

Although AI presents novel possibilities to improve clinical care of cognitive impairment and related disorders, the integration of AI into standardized healthcare practice may be hindered by ethical challenges, including researcher, clinician, and patient understanding of technological bias that could exacerbate health inequities.^9^ There are also concerns specific to the context of cognitive impairment; for example, breaches of patient privacy and/or the occurrence of a false positive diagnosis of cognitive impairment could result in a range of social and legal consequences related to insurance coverage, decisions about independent living (eg, driving a vehicle), and risks to mental wellbeing, such as stigma or shifting perceptions of personal identity.^10,11^ The ability for clinicians to address such challenges is constrained by a lack of consensus of “best practices” guidelines for AI clinical integration, which can act as a barrier to AI adoption in healthcare settings.^12^

Patients’ perspectives on benefits, harms, and consent can play an important role in establishing ethical guidelines and practical approaches to the use of AI-driven tools in clinical care. Widespread patient acceptance of novel healthcare technology hinges on approaches to AI use in the clinic that align with the preferences of patients and mitigate potential harms, as perceived by patients and their families. While there is an extensive body of literature on the ethical considerations of AI in clinical practice,^4,13,14^ we found few studies that explored patient perspectives and none that investigated specifically patients’ views on AI use for cognitive impairment detection. To address this gap, we conducted qualitative interviews with primary care patients, focusing on the use of AI to synthesize data from EMRs and audio recordings of patient speech. We aimed to gather perspectives from a racially and educationally diverse group of patients to ensure representation.

## 2 | METHODS

### 2.1 | Sample

Participants were recruited from a parent study with the objective of developing automated cognitive impairment screenings. The AI tool was a ML model that employed EMR data and audio recordings of patients during primary care visits. For the derivation set, participants of the parent study were recruited from three hospital-based primary care teaching practices in the Mount Sinai healthcare system in New York City from August 2020 through December 2021. Eligible patients were 55 years and older, spoke English, were able to provide informed consent in English, and had no diagnosis of mild cognitive impairment or dementia in the problem list or medical history of their medical record. Individuals who consented to be contacted for future research served as the recruitment pool for the current study. We selected a purposeful sample to achieve a diverse representation in age, sex, race, and ethnicity (Black, White, and Latinx). A letter was mailed to prospective participants describing the study followed by a recruitment call from a research coordinator. We ceased recruitment when saturation was met. The Institutional Review Board of the Ichan School of Medicine at Mount Sinai approved all study procedures.

### 2.2 | Data collection and analysis

This study employed a qualitative descriptive design with the aim of presenting a comprehensive summary of findings with minimal theorization.^15^ Qualitative description is a well-suited methodology for studies that explore perceptions and concerns about specific medical phenomena among patients and providers.^15–17^ We conducted individual qualitative interviews using a semi-structured interview guide (Supporting Information A) that included open-ended questions to elicit participant narratives stemming from their lived experiences. The interview set the context for the discussion by assessing participants’ experiences with people with dementia and their perceptions of dementia treatments and screening. We then queried their understanding of AI and provided a basic description of how AI could be employed in clinical settings to support clinicians’ work. To ground their understanding of screening, we used colon cancer screening, which is commonly done in persons ages 50 years and older, as an example. Participants were then presented with two hypothetical scenarios for automated cognitive impairment screening in primary care that could be used to aid doctors in identifying cognitive impairment for early intervention. In the first scenario, a ML algorithm embedded in the EMR would use data from that source to identify patients with cognitive impairment, while the second, also embedded in the EMR, would employ audio recordings of patients’ speech during their visit with a doctor to identify cognitive impairment. We then asked participants for their thoughts about the potential benefits and harms of these screening modalities and their views on consent from patients, including whether consent for the use of such tools should be required and how it should be obtained. We used an infographic to promote discussion about potential processes for consent (Supporting Information B). Participants were encouraged to ask questions throughout the interview. We developed the interview guide using a review of the literature and iterative revisions among experts on the study team. We then tested the interview guide for question comprehension and to ensure that the questions would elicit data relevant to the study goals. Interviews were conducted via Health Insurance Portability and Accountability Act (HIPAA)-compliant Zoom video conference (Zoom Video Communications, San Jose, CA, USA) or in person. We reached thematic saturation at 31 interviews, at which point no new themes or insights emerged from the data.

Interviews were recorded, professionally transcribed, and then uploaded to ATLAS.ti qualitative data analysis software (ATLAS.ti Scientific Software Development GmbH, Berlin, Germany). Data were analyzed using content analysis. Two team members independently conducted preliminary coding of two transcripts using codes derived from the interview guide and existing literature. Codes that emerged within the data were identified concurrently and integrated into an updated codebook. Three team members then redundantly coded two additional transcripts. The codebook was finalized through team member agreement, and then all transcripts were coded independently by three analysts who met to resolve discrepancies. Following the completion of coding and code checks, analysts wrote thematic memos summarizing findings of three to four codes grouped into broader themes (eg, “ethical considerations” was based on the codes “consent procedures,” “emotional response to screening: moral dimensions,” and “clinician mistrust”). Memos were refined and integrated into a coherent narrative, which was then reviewed by the wider study team to validate findings. Dominant themes were determined according to the frequency and quality of participant responses. The quality of responses was based on the richness and nuance of participant accounts, as well as the potential to expand the extant literature.

## 3 | RESULTS

We conducted interviews with 31 individuals, with 16 interviews conducted via video conference. Interviews ranged from 30 to 120 minutes in duration. The mean age of participants was 67 years (range, 59 to 85 years); 18 participants (58%) were women, 18 (58%) were Black, and seven (23%) were Latinx (Table 1).

### 3.1 | Perceived benefits of implementing automated cognitive impairment screening using AI

Participants did not have a uniform interpretation of what constituted AI screening or the scope of its role in the diagnostic process. Some thought of AI as a tool that could assist the doctor – “[The doctor]’s using the AI to help him out” (*61 years, male, other race, vocational or technical school*). Others described AI as an autonomous, decision-making entity. In several cases, participants conflated the screening process with treatment for cognitive impairment. Despite varied interpretations of how AI functions, participants reported a range of benefits from AI screening for both patients and clinicians. Two major themes emerged: (1) improved outcomes for detecting and diagnosing cognitive impairment and (2) value of results to make decisions about treatment and care (Table 2).

#### 3.1.1 | Improved outcomes for cognitive impairment detection and diagnosis

Participants reported that AI testing would improve accuracy and decrease human error in detecting cognitive impairment. Some felt that AI could yield more accurate and objective results than screening done by a human being and potentially help prevent misdiagnosis. Although select participants commented on the possibility that information could get “misread” by the computer, most participants who discussed misdiagnosis attributed it to clinician errors, such as bias, oversight, or misreading of diagnostic indicators. “…sometimes physicians don’t see [certain symptoms] that people who are taking care of people that have these illnesses do see” (*69 years, female, Black, some college*). AI screening was viewed as a potential mechanism for preventing or mitigating clinician errors because the technology did not rely on human judgment and would not be subject to human mistakes, making it “smarter than a human” *(60 years, male, other race, some college)*.

I think a machine would be…nonjudgmental… It would just [provide] an objective point of view, not someone who would know me personally. So, I would feel more confident in the machine actually

(*64 years, female, White, associate degree*).

Participants also discussed ways that AI technology might improve patient access and acceptance of cognitive impairment screening procedures. Some described this in terms of improved workflow efficiency for healthcare providers and reduced screening time, possibly enhancing the feasibility of integrating standardized cognitive impairment screening into primary care practice. Others mentioned that AI screening might reduce patient expenses, stressors, and wait times associated with specialist referrals and invasive forms of testing.

Make life easy. There’s a lot of tests. They are sometimes unnecessary and doesn’t agree with the patient. So, maybe avoiding that and going straight to what should be done is a lot easier. It’s like they don’t have to guess anymore and put people through anything. It will save also health insurance and money on patient’s pocket

(*61 years, female, Black, high school graduate or GED*).

#### 3.1.2 | Use of screening results to make decisions about treatment and care

Participants reported that AI screening could support patients’ decision-making about treatment and care upon receiving a positive detection or diagnosis of cognitive impairment. For those who spoke about how AI screening results might inform present healthcare decision-making, their discussions often centered on the assumption that screening results would provide them with accurate and medically actionable information.

Because if something is going on with me, with my cognitive behavior or my cognitive intelligence … I would like to know before it gets worse and what I need to do or what I would hope I could get to keep it stabilized or improved

(*77 years, female, Black, some college*).

Participants who discussed future decision-making highlighted practical considerations, such as purchasing health insurance. They also described using screening results to inform care coordination, particularly with family members, with considerations of living arrangements, psychosocial support, and accident prevention.

For the future [AI screening] might be helpful. If something does come down the pike, then I could say, ‘Well, yes, I need help … And maybe get covered by insurance for something I might need

(*64 years, female, White, associate degree*).

### 3.2 | Negative perceptions of automated cognitive impairment screening using AI

While participants described more benefits than potential harms from using AI for screening, they also expressed concerns about possible negative effects (Table 3).

#### 3.2.1 | Risk of incorrect information and compromised data security

Some participants shared concerns regarding potential errors in AI technology that could lead to misdiagnosis – particularly if there were an over-reliance on automated screening in the absence of clinical evaluation. Due to such concerns, participants expressed a strong preference for clinician use of AI-based screening to prompt further evaluation rather than relying on it for diagnosis.

[You can’t just read the AI and say], “Oh, I think you got dementia.” Boom. I don’t think it should be done like that. I think that physicians need to kind of step back and take a look at your patient. I know you have a lot of them, but the thing in healthcare is doing healthcare

(*69 years, female, Black, some college*).

Participants also expressed concerns about potential technological problems that could result from implementation – for example, data breaches and loss of confidentiality that might occur if hackers were able to access clinical information. Stigmatization and changes in insurance status or coverage were commonly cited as potential ramifications if insurance companies, acquaintances, or other actors were to learn of screening results and diagnoses without permission.

You could hack my medical records … I think the only harm that can come to that is denial of insurance. But there are people with conditions that they see as stigmatized. Maybe there’s somebody who sees dementia stigmatizing, or humiliating … So, that could be a problem … It’s a big institution protecting your data. We all know that there are big institutions, banks, Anthem insurers, right, “that have been hacked …”

(*67 years, male, White, post-graduate degree*).

#### 3.2.2 | Mental health distress

The psychological consequences of automated screening were at the forefront of participants’ discussions of potential harms. Some participants shared that they may not want to know the results of automated screening for cognitive impairment. Receiving a positive screening result for a condition they perceived themselves as having limited control over would increase anxiety about the future. Relatedly, others mentioned that positive screening results could potentially cause or exacerbate symptoms of mental health distress such as depression, anxiety, or social withdrawal.

I think worry and anxiety and defensiveness … people get scared and then people have this hanging over them. “Oh, I’m deteriorating,” and so, yes, I could see that that would make your life less optimistic or sadder…

(*67 years, male, White, post-graduate degree*).

### 3.3 | Participant preferences for consent and disclosure of results

Participants expressed preferences for how healthcare practitioners might obtain consent from patients for automated cognitive impairment screening (Table 4). Most participants agreed that information about screening should be provided at the beginning of a visit, with many preferring the option to opt-in or opt-out before screening occurs.

I would feel like I was being violated without my knowledge … that would be wrong. I mean, I may accept it if I was pre-notified that this was going to be taking place, but this is like, I feel like I’ve been ambushed

(*77 years, female, Black, some college*).

When asked about a consenting process, most participants said they would prefer a physical form to review while the information is read aloud. Then they would sign and have a copy of the form for their records.

Explain [the consent form] to me verbally because I have no patience for reading. So, if you tell me what it is, I can scan through the paperwork. I can read it as you’re talking and I can just sign it or just give you a verbal okay

(*64 years, male, Native American/Alaskan Native, high school graduate or GED*).

Participants emphasized the importance of transparency during the consent process along with other screening procedures. Some participants noted they would be against screening if the clinician were not forthright about the process and sharing of results. They expressed a desire for clear and accurate information on how the screening is performed, how the clinician interprets the results, and the objectivity of the process. For example, “I would want to know what they looked at and how they [the clinician] came to the conclusion” *(66 years, female, Black, 4-year college graduate)*.

Some participants emphasized that they would be more likely to accept the screening if they understood what the procedure entailed.

People fear the unknown because they’re not explained to [about] how to do it. That’s the problem. Sometimes, you don’t tell a person the reason why you want to do this and they get nervous. “Oh, no, I don’t want to do it because I think it’s going to hurt me.” But if you explain to them, people would do the screening

(*60 years, male, other, some college*).

A trusting relationship with the clinician was an important factor in how participants assessed automated cognitive impairment screening and accepted screening procedures. “… if the information just went to my doctor and my doctor talked to me about it, that would be good because I trust my doctor and I feel comfortable with her, and I just wouldn’t want it exposed all over … to insurance companies or anything that can harm me” (*64 years, female, White, associate degree*).

Some participants also believed that involving a family member or friend in the consent process would enable a greater reception of screening and maximize its benefits. They described how having a support person present might help avoid miscommunication due to language differences or disparities in education. Such persons could also provide important information and observations about the patient’s daily life that might help the clinician in contextualizing screening results more effectively.

Sometimes information gets skewed … and misread, and that could be a problem. I think that would be harmful. But a patient or a patient care advocate would be able to recognize … if something’s not right and ask for a reassessment of the information, or a second screening

(*66 years, female, Black, post-graduate degree*).

## 4 | DISCUSSION

This study found that patient participants reported generally positive impressions of automated cognitive impairment screening. Participants believed that AI technology could improve diagnostic accuracy and avoid human error in evaluation and diagnosis while also improving clinical efficiency for clinicians. Yet they also expressed concerns such as risk of misdiagnosis, loss of confidentiality, and psychological distress from learning of detected cognitive impairment. Some participants believed that automated screening should only be used to supplement other evaluations. Moreover, they underscored the importance of transparency in the use of such a screening modality. They suggested that informed consent was necessary prior to screening and that results should be provided with clear explanations about accuracy and how the screening works. Based on findings from this study, we highlighted the significant ethical challenges facing the use of AI in clinical care, and for cognitive impairment specifically. Importantly, our use of patient input goes beyond current literature addressing the ethics of AI use in healthcare.

One key concern often cited by ethicists and others is the potential for biases in ML algorithms.^18^ ML models may be trained on historical data or on current data that reflect existing societal biases or disparities in healthcare. Without correcting such biases, inaccurate assessments of patients may occur, potentially exacerbating disparities. Consistent with this concern, most patients in this study recognized that errors of accuracy and reliability of the technology were possible despite expressing a limited understanding of how AI functions. Before technologies like automated cognitive impairment screening are implemented, they must be evaluated to avoid the propagation of biases. Clinicians using these technologies should be prepared to address concerns about the accuracy of data generated.

Study participants also expressed considerable concerns about privacy, including loss of confidentiality and data breach risks, and consequences such as new barriers to insurance coverage and stigmatization with diagnosis. As with any sensitive personal health information, data generated from ML models for screening must be secured to protect privacy and prevent breaches. This is a particular concern among minoritized patients who may have a historical distrust of healthcare systems.^19^ Patients must be informed about how their data will be used and should be given the chance to consent to their use for screening purposes.

Consistent with previous studies on the emotional responses of patients to threatening health information,^20,21^ study participants frequently cited concerns about psychological distress that might be generated after the detection of cognitive impairment through automated screening. Fortunately, such distress might be mitigated through established clinical practices such as pre-test counseling, post-test counseling and support, the provision of informational resources, and supportive communication strategies by clinicians during risk disclosure sessions with patients and their loved ones.^20,22,23^

A prevailing theme that emerged from participants’ observations and concerns centers on the necessity of clear communication. This may present one of the most significant challenges for healthcare providers. Clear and transparent communication requires clinician literacy in AI-driven tools, a potential barrier due to “black box” issues and the evolving nature of the technology. Additionally, clinicians may be limited in their capacity to allocate sufficient time to explain the technological functions, accuracy, security measures, and interpretation of results. Such information sharing may need to occur during an informed consent process, as many patients in this study believed that written informed consent should be obtained before implementing automated screening for cognitive impairment or generating data from such screening. Clinicians often have limited time to address issues they deem essential for patients’ health during a consent process.^24^

Resources and training are needed to support clinicians as the potential for clinical integration of AI-driven tools emerges. Although clear guidance is still lacking, ongoing advancements in responsible AI practices have begun to make headway in improving pathways for clinicians to access and engage AI technologies.^25–27^ Explainable AI (XAI) programs, for example, may aid clinicians in generating effective explanations about the decisions, actions, and performance of ML models.^28^ Establishing guiding principles and standards for the development and use of AI tools may also bolster clinician trust in and acceptability of new AI technologies in clinical settings by promoting “best practices” and ethically supportable approaches to the healthcare application of AI models, including guidance on transparent data handling, adaptability of AI approaches to the local population, and shared decision-making between clinicians and their patients.

### 4.1 | Limitations

A major strength of this study is its focus on patients’ perspectives regarding the use of AI in healthcare, specifically for cognitive impairment screening, and its inclusion of a diverse sample of patients, including older Black and Latinx participants. As a qualitative study from a single site with a small sample size, there are limitations. Study results may not be generalizable to patients in other settings or with different sociodemographic backgrounds. Notably, we did not include non-English speakers. Future research on the acceptability of AI in healthcare, and dementia screening in particular, should aim to include a broader demographic spectrum of patients, including language diversity. The scenarios and questions we posed for study participants were hypothetical, and patients’ views on hypothetical situations may differ from their views based on lived experiences. However, the purpose of this study was to assess participant perspectives that could guide future implementation of the described technology. Relatedly, we did not discuss examples of automated analysis of patient data to inform clinical decision-making that is commonly used in health systems, such as EMR-embedded clinical risk prediction models for conditions like cardiovascular disease. Awareness of such automated modeling among study participants might have influenced their views on automated cognitive impairment screening, though the directionality of the effect is unclear.

## 5 | CONCLUSION AND IMPLICATIONS

Given rapid advances in AI, the high performance of early models of automated cognitive impairment detection, and increasing reasons to screen, widespread clinical implementation of automated cognitive impairment screening may become a reality in coming years. Data from this study suggest next steps researchers and health systems could take to pave the way for the ethical and successful implementation of such screening. These include developing language and communication strategies to ensure that patients understand how such technology works when they have questions about it and providing clinicians the training and resources to achieve this objective. Part of a successful communication strategy should include anticipating and developing language to address patients’ and caregivers’ concerns around issues like accuracy of the technology, data privacy, and stigmatization. Research should also further explore the consent process. While health systems might have the right to use EMR data to support clinical decision-making without obtaining patient consent for the specific purpose,^29^ some patients, as indicated by our findings, might strongly disagree with such implied consent, at least for cognitive assessment. Without consent, patients’ trust in their provider could be at risk. Thus, testing candidate consent processes for feasibility and acceptance among patients and providers would be important preparatory work. Finally, screening could induce distress among patients with newly identified cognitive impairment. Health systems should be prepared with supportive language and resources to address patient concerns in such circumstances.

## Supplementary Material

Artificial intelligence as a tool for cognitive impairment screening: Patient perspectives about benefits and limitations Supplemental A_ Interview Guide

Artificial intelligence as a tool for cognitive impairment screening: Patient perspectives about benefits and limitations_ Supplemental B Consent Infographic

Additional supporting information can be found online in the Supporting Information section at the end of this article.

## Figures and Tables

**TABLE 1:** Participant demographics.

Variable	*n*	Percentage (%)
Gender		
Male	13	41.9
Female	18	58.1
Race		
Black/African American	18	58.1
White/Caucasian	7	22.6
Native American/Alaskan Native	2	6.5
Other	3	9.7
Refused	1	3.2
Hispanicor Latino		
Yes	7	22.6
No	24	77.4
Highest level of education		
Some high school	7	22.6
High school graduate or GED	4	12.9
Vocational or technical school	1	3.2
Some college	6	19.4
Associate degree	1	3.2
4-year college graduate	5	16.1
Post-graduate degree	5	16.1
Unspecified	2	6.5
Age		
50 to 59	3	9.7
60 to 69	17	54.8
70 to 79	6	19.4
80 to 89	5	16.1

**TABLE 2:** Participant quotations about benefits of implementing AI tools for cognitive impairment screening.

**Subtheme 1: Improved outcomes for detection and diagnosis**
*Artificial intelligence is a computer that is smarter than a human being. It can make decisions for you that could tell you if something is right or wrong and find the answer for the right questions. (60 years, male, other, some college)*
*The computer has more technology [than the doctor]. The doctors don’t know, the computer knows…It’s a computer doing it. So, that’s all they can say is the computer came up with it. (66 years, female, Black, 4-year college graduate)*
*A lot of doctors make mistakes. A lot of mistakes. Maybe they need assistance. This would be a good thing. A lot of doctors make a lot of misdiagnoses. A lot of doctors make mistakes. Maybe it’s time to get help. (61 years, female, Black, high school graduate or GED)*
*… humans can do misdiagnosis or stuff I think the machine, not having the emotional -the preconceptions of some kind of prejudice or some kind of opinion might be a little more mathematical and more logical than a human being. (64 years, male, Native American/Alaskan Native, high school graduate or GED)*
*I have a pretty big medical file, so that’s going to be a long process, but I’ve had experience with a doctor, say … maybe they wrote stuff down that instead of taking responsibility for things they might have said to me or condescended, they said it’s because I’m anxious or it’s my age. So, that kind of thing … It’s not all one sided, I think. (64 years, female, White, associate degree)*
*“… sometimes physicians don’t see [certain symptoms] that people who are taking care of people that have these illnesses do see. (69 years, female, Black, some college)*
*[AI screening] presents in my mind an argument for … making it part of routine data collection, just as - I think it’s so much more precise if AI successfully does this, it could be so much more precise than “so, how are you doing?” (67 years, male, White, post-graduate degree)*
*I just think that it makes life much easier for the practitioner, much easier to diagnose … he can either do the analysis from the AI or do his personal analysis on it so he can determine which one is a little bit - which one is accurate. (61 years, male, other, vocation or technical school)*
*You can diagnose it much faster. (75 years, male, White, post-graduate degree)*
*It could save a lot of time and energy for both the doctor and the patient. (64 years, male, Native American/Alaskan Native, high school graduate or GED)*
*[AI screening] would take the healthcare level higher because, not only now your primary care physician dealing with your physical, but your primary care begins to take some responsibility for your mental, your cognitive thinking … it would give the primary care physician an assessment tool so that they could refer the patient to a specialist which would further help their treatment. (66 years, female, Black, post-graduate degree)*
*I think it might be better because you get a result or finding quicker which will help, instead of waiting 2 to 3 months … Instead of making so many trips and have to wait there, you have to go see the specialist and come back. (67 years, female, Black, some high school)*
*I would agree to that because I don’t think there’s any kind of danger in that because they’re not touching you at all … I’m a chicken with blood tests … It’s better than getting - like drawing bloods and taking all kind of X-rays and tests, which the radiology and pumping all stuff into your blood, to your blood system. (66 years, female, Black, 4-year college)*
**Subtheme 2: Decision-making about treatment and care**
*Because if something is going on with me, with my cognitive behavior or my cognitive intelligence … I would like to know before it gets worse and what I need to do or what I would hope I could get to keep it stabilized or improved. (77 years, female, Black, some college)*
*If my doctor could detect early deterioration… with this information, she could refer me to the doctors I need to see and then they can tell me, “Well, you can do this and then something, or you could do that without medication.” That’s just my preference. “Let’s see what we can do to push off the Alzheimer’s, the dementia, whatever, and let’s see if we can just keep you going” … That’s why it would be good. It would help me have a better quality of life for a longer period of time. (66 years, female, declined to report race/ethnicity, 4-year college graduate)*
*Well, that could help to look down the road if there’s any patterns of past screenings of other people could help to say, “Well, this person might have dementia 5 years from now” or “This person is showing cognitive results of pre-Alzheimer’s.” (69 years, female, Black, some college)*
*It will be a benefit for the person if, later on in life, you would know if you’re going to have dementia and stuff like that. (64 years, female, Black, high school graduate or GED)*
*… for the future [AI screening] might be helpful. If something does come down the pike, then I could say, “Well, yes, I need help, and look, it started here. It’s progressed,” or whatever it is. And maybe get covered by insurance for something I might need. (64 years, female, White, associate degree)*
*I guess I want to know to let my siblings all know because they would be the ones trying to help me out. (66 years, female, Black, 4-year college graduate)*
*I’m here to - God got to be here to take care of my little 10-year-old daughter. Like I said, I’m a single dad, and I need to know everything and anything that’s going on [with my health]. (64 years, male, Black, some high school)*
*… people can plan for their care depending upon the angle of their decline. (82 years, male, White, post-graduate degree)*
*I think it would be valuable to know [screening results] to plan, like plan in the future, maybe delay it a little bit with medications and plan. Do I need a nursing home? Do I want to kill myself? I know that’s being a little dramatic, but I don’t want to wind up walking the streets, screaming at people because I have dementia. You know what I’m saying? Or getting lost somewhere. Because I’m pretty much independent, so I wouldn’t want that to happen. It’s kind of a scary thing, but maybe I wouldn’t want to add another drug. (64 years, female, White, associate degree)*

**TABLE 3:** Participant quotations about negative perceptions of implementing AI tools for cognitive impairment screening.

**Subtheme 1: Risk of Incorrect Information and Compromised Data Security**
*How safe is the information?… like your credit, your social security, your address, you wouldn’t want all those things out in the open. Anyone can just grab and do whatever they want with it. People are doing that to people every day (59 years, male, Black, post-graduate degree)*
*I guess the biggest part about it is just fear of what the machine is going to do with your information. Is it going to call your mother, or going to call your daughter, or your wife, and tell them the secrets? (64 years, male, Native American/Alaskan Native, high school graduate or GED)*
*You could hack my medical records … I think the only harm that can come to that is denial of insurance. But there are people with conditions that they see as stigmatized. Maybe there’s somebody who sees dementia stigmatizing, or humiliating, or they’re embarrassed by it … So, that could be- it could be a problem … It’s a big institution protecting your data. We all know that there are big institutions, banks, anthem insurers, right, that have been hacked, and so …” (68 years, male, White, post-graduate degree)*
*My one thing that comes to mind is that what if - because these are computers - what if the wrong information gets out there, and I really don’t have any deterioration, and my doctor gets this information, or the computer misreads it, that’s my own - that’s the only thing I’m concerned about is misinformation. (66 years, female, declined to report race/ethnicity, 4-year college graduate)*
*I think there’s a potential, I’ll put it this way, societal harm in the premature use of algorithms to deal with individuals. (82 years, male, White, post-graduate degree)*
*Something might go wrong with technology and something might happen. (66 years, female, other, high school graduate or GED)*
*Sometimes information gets skewed and that could be - sometimes information gets misread, and that could be a problem (66 years, female, Black, post-graduate degree)*
*There’s potential for misuse and prejudice and everything else, and for being wrong. We can’t let the AI machine decide, “Oh, well, you are getting a worker in your house because you’re so cognitively impaired,” and it could be assuming that from language, it could be [skewed] (67 years, male, White, post-graduate degree)*
*I think that if it’s done properly in a proper way and I say, “Oh, I think you got dementia.” Boom. I don’t think it should be done like that. I think that physicians need to kind of step back and take a look at your patient. I know you have a lot of them, but the thing in healthcare is doing healthcare and that’s all of it. (69 years, female, Black, some college)*
*I think that if it’s done properly in a proper way and I say, “Oh, I think you got dementia.” Boom. I don’t think it should be done like that. I think that physicians need to kind of step back and take a look at your patient. I know you have a lot of them, but the thing in healthcare is doing healthcare and that’s all of it (69 years, female, Black, some college*).
**Subtheme 2: Mental health distress**
*I think it’s a terrible concern that people could commit suicide or just become mentally, just, depressed and not function, and ruin the remaining years that they do have. (67 years, male, White, post-graduate degree)*
*I’m not worried about the doctor, but it’s just … it would scare me, because then I would be so anxious. When is it going to happen? When is it going to happen? That’s the only reason why I wouldn’t want to know, and I probably would worry myself. If I can’t remember something, maybe I can’t remember a word to tell you and then I’m out, and I’ll be like, “Oh my God, my dementia starting already.” You understand? That’s the part that I don’t want to know. (66 years, female, Black, 4-year college graduate)*
*I think worry and anxiety and defensiveness, right? I think those are - people get scared and then people have this hanging over them. “Oh, I’m deteriorating,” and so, yes, I could see that that would make your life less optimistic or sadder, because you figure you have this. Like somebody who learns that they have Huntington’s Chorea, and you know you’re going to deteriorate if you have the full genetic inheritance of that. (67 years, male, White, post-graduate degree)*
*Interviewer: If your doctor were to ask you if you would like to be screened for cognitive impairment, what would be some of your thoughts on that?* *Respondent: Okay. The next place would be the funny farm. I’ve lost it. “Uh-oh. I better watch my steps, man, he’s asking me this question for some reason. I better watch out.” Those are some thoughts I would probably have. (59 years, male, Black, post-graduate degree)*
*I think most people would find it too anxiety or too unhappiness provoking … anxiety and then sadness and depression loosely define … Yes, some vulnerable people might be triggered into paranoid episodes. (82 years, male, White, post-graduate degree)*
*I don’t see any harm unless, later on, something [results] comes back and tells me I’ve got an incurable disease. That would be harmful. That would wreck my brain. That would send me into a tailspin, but I would pray that God would take it away from me and let me live out my days. (69 years, female, Black, some college)*
*Well, if it got to my insurance company, for instance, I mean, maybe there’s going to be a charge, there’s probably going to be a code for billing. But from my insurance company, I just - or maybe something would come up and then I find out it’s not covered. Just the stress of affecting, just something going wrong with my medical coverage or prescription coverage would be really disastrous for me. (64 years, female, White, associate degree)*

**TABLE 4:** Participant quotations about preferences for consent and disclosure of results.

*It would be … I would feel like I was being violated without my knowledge. I would feel that that would be wrong. I mean, I may accept it if I was pre-notified that this was going to be taking place, but this is like, I feel like I’ve been ambushed, to my knowledge. I feel that that would be wrong. (77 years, female, Black, some college)*
*Explain it [the consent form] to me verbally because I have no patience for reading. So, if you tell me what it is, I can scan through the paperwork. I can read it as you’re talking and I can just sign it or just give you a verbal okay. (64 years, male, Native American/Alaskan Native, high school graduate or GED)*
*People fear the unknown because they’re not explained to them how to do it. That’s the problem. Sometimes, you don’t tell a person the reason why you want to do this and they get nervous. “Oh, no, I don’t want to do it because I think it’s going to hurt me.” But if you explain to them, people would do the screening. (60 years, male, other, some college)*
*Well, if the information just went to my doctor and my doctor talked to me about it, that would be good because I trust my doctor and I feel comfortable with her, and I just wouldn’t want it exposed all over … to insurance companies or anything that can harm me. (64 years, female, White, associate degree)*
*I think one way [to approach consent] would be to work with the caregiver, whether it’s a family member, or a home attendant, however it works for that particular patient. I think going through them would be an effective means of communication. (66 years, female, Black, post-graduate degree)*
*I will see what - we can do it, me and my daughter. She will come with me and we can sit down and listen … I do a lot of English but my Spanish is much … I work with my daughter … Whenever I go to the doctor, she’ll go with me, or the doctor call her and they speak to her. (83 years, female, Black, unspecified education level)*
*Sometimes information gets skewed and that could be - sometimes information gets misread, and that could be a problem. I think that would be harmful. But a patient or a patient care advocate would be able to recognize - should be able to recognize if something’s not right and ask for a reassessment of the information, or a second screening. (66 years, female, Black, post-graduate degree)*

## Highlights

- Key point 1. Patient participants reported generally positive perspectives on a potential AI system for automated cognitive impairment screening, although they also expressed concerns about the risk of misdiagnosis, loss of confidentiality, and psychological distress from learning of detected cognitive impairment
- Key point 2. Participants’ concerns about the potential harms of AI screening tools underscore the need for clear and transparent communication about how results are generated and interpreted. Clinicians need training and other forms of structural support to improve their literacy in AI-driven tools and their ability to integrate adequate approaches to information sharing into clinical practice.
- Key point 3. Most patients in this study recognized that errors of accuracy and reliability of the technology were possible. Before technologies like automated cognitive impairment screening are implemented, they must be evaluated to avoid the propagation of biases. Clinicians using these technologies should be prepared to address concerns about the accuracy of the data generated.

## RESEARCH IN CONTEXT

1. **Systematic review**: We reviewed the literature through a systematic search in PubMed and other related sources on the use of AI as a tool in clinical settings, including ethical considerations. A scoping review (in preparation), which was part of the parent study, further supported this review. While there is an extensive body of literature on the ethical considerations of AI in clinical practices, we found few studies that explored patient perspectives and none that interrogated specifically patients’ views on AI use for cognitive impairment detection or diagnosis.
2. **Interpretation**: To address this gap, we conducted qualitative interviews with primary care patients, focusing on the use of AI to synthesize data from EMRs and audio recordings of patient speech.
3. **Future directions**: This study sheds light on patients’ perspectives on the use of AI as a screening tool for cognitive impairment. Clinical guidelines that consider patients’ perspectives are needed to align the implementation of AI tools with patient values and views on benefits and harms. An understanding of patients’ perspectives is crucial for developing ethically supported clinical practices and for increasing patient access to screening for cognitive impairment in clinical settings.

## References

[1] Graham SA, Lee EE, Jeste DV, Artificial intelligence approaches to predicting and detecting cognitive decline in older adults: a conceptual review. Psychiatry Res. 2020;284:112732. doi: 10.1016/j.psychres.2019.112732 31978628 PMC7081667

[2] Holsinger T, Plassman BL, Stechuchak KM, Burke JR, Coffman CJ, Williams JW. Screening for cognitive impairment: comparing the performance of four instruments in primary care. J Am Geriatr Soc. 2012;60(6):1027–1036. doi: 10.1111/j.1532-5415.2012.03967.x 22646750

[3] Gervasi SS, Chen IY, Smith-McLallen A, The potential for bias in machine learning and opportunities for health insurers to address it: article examines the potential for bias in machine learning and opportunities for health insurers to address it. Health Aff (Millwood). 2022;41(2):212–218. doi: 10.1377/hlthaff.2021.01287 35130064

[4] Goirand M, Austin E, Clay-Williams R. Implementing ethics in healthcare AI-based applications: a scoping review. Sci Eng Ethics. 2021;27(5):61. doi: 10.1007/s11948-021-00336-3 34480239

[5] Hussein KI, Chan L, Van Vleck T, Natural language processing to identify patients with cognitive impairment. medRxiv. 2022.02.16.22271085. Preprint posted online February 17, 2022. doi: 10.1101/2022.02.16.22271085

[6] Saheb T, Saheb T, Carpenter DO. Mapping research strands of ethics of artificial intelligence in healthcare: a bibliometric and content analysis. Comput Biol Med. 2021;135:104660. doi: 10.1016/j.compbiomed.2021.104660 34346319

[7] Dublin S, Greenwood-Hickman MA, Karliner L, The electronic health record Risk of Alzheimer’s and Dementia Assessment Rule (eRADAR) Brain Health Trial: protocol for an embedded, pragmatic clinical trial of a low-cost dementia detection algorithm. Contemp Clin Trials. 2023;135:107356. doi: 10.1016/j.cct.2023.107356 37858616 PMC11244615

[8] Coley RY, Smith JJ, Karliner L, External validation of the eRADAR risk score for detecting undiagnosed dementia in two real-world healthcare systems. J Gen Intern Med. 2023;38(2):351–360. doi: 10.1007/s11606-022-07736-6 35906516 PMC9904522

[9] Challen R, Denny J, Pitt M, Gompels L, Edwards T, Tsaneva-Atanasova K. Artificial intelligence, bias and clinical safety. BMJ Qual Saf. 2019;28(3):231–237. doi: 10.1136/bmjqs-2018-008370 PMC6560460 30636200

[10] Cornett P, Hall J. Issues in disclosing a diagnosis of dementia. Arch Clin Neuropsychol. 2008;23(3):251–256. doi: 10.1016/j.acn.2008.01.001 18299184

[11] Rosenfeld L, Torous J, Vahia IV. Data security and privacy in apps for dementia: an analysis of existing privacy policies. Am J Geriatr Psychiatry. 2017;25(8):873–877. doi: 10.1016/j.jagp.2017.04.009 28645535

[12] Ahmed MR, Zhang Y, Feng Z, Lo B, Inan OT, Liao H. Neuroimaging and machine learning for dementia diagnosis: recent advancements and future prospects. IEEE Rev Biomed Eng. 2019;12:19–33. doi: 10.1109/RBME.2018.2886237 30561351

[13] Chen IY, Pierson E, Rose S, Joshi S, Ferryman K, Ghassemi M. Ethical machine learning in healthcare. Annu Rev Biomed Data Sci. 2021;4(1):123–144. doi: 10.1146/annurev-biodatasci-092820-114757 34396058 PMC8362902

[14] Gerke S, Minssen T, Cohen G. Ethical and legal challenges of artificial intelligence-driven healthcare. Artificial Intelligence in Healthcare. Elsevier; 2020:295–336. doi: 10.1016/B978-0-12-818438-7.00012-5

[15] Sandelowski M. Whatever happened to qualitative description? Res Nurs Health. 2000;23(4):334–340. doi: 10.1002/1098-240X(200008)23:4〈334::AID-NUR9〉3.0.CO;2-G 10940958

[16] Smeltzer SC. The concerns of pregnant women with multiple sclerosis. Qual Health Res. 1994;4(4):480–502. doi: 10.1177/104973239400400409

[17] Geller G, Holtzman NA. A qualitative assessment of primary care physicians’ perceptions about the ethical and social implications of offering genetic testing. Qual Health Res. 1995;5(1):97–116. doi: 10.1177/104973239500500107

[18] Nazer LH, Zatarah R, Waldrip S, Bias in artificial intelligence algorithms and recommendations for mitigati. PLOS Digit Health. 2023;2(6):e0000278. doi: 10.1371/journal.pdig.0000278 37347721 PMC10287014

[19] Griffith DM, Bergner EM, Fair AS, Wilkins CH. Using mistrust, distrust, and low trust precisely in medical care and medical research advances health equity. Am J Prev Med. 2021;60(3):442–445. doi: 10.1016/j.amepre.2020.08.019 33208267 PMC7902381

[20] Guan Y, Roter DL, Wolff JL, The impact of genetic counselors’ use of facilitative strategies on cognitive and emotional processing of genetic risk disclosure for Alzheimer’s disease. Patient Educ Couns. 2018;101(5):817–823. doi: 10.1016/j.pec.2017.11.019 29203084 PMC5911203

[21] Di Mattei VE, Carnelli L, Bernardi M, Coping mechanisms, psychological distress, and quality of life prior to cancer genetic counseling. Front Psychol. 2018;9:1218. doi: 10.3389/fpsyg.2018.01218 30061853 PMC6055025

[22] Ellington L, Kelly KM, Reblin M, Latimer S, Roter D. Communication in Genetic Counseling: cognitive and emotional processing. Health Commun. 2011;26(7):667–675. doi: 10.1080/10410236.2011.561921 21660793

[23] Greer JW, Milam RA, Eggers PW. Trends in use, cost, and outcomes of human recombinant erythropoietin, 1989–98. Health Care Financ Rev. 1999;20(3):55–62. 10558020 PMC4194623

[24] Caverly TJ, Hayward RA. Dealing with the lack of time for detailed shared decision-making in primary care: everyday shared decision-making. J Gen Intern Med. 2020;35(10):3045–3049. doi: 10.1007/s11606-020-06043-2 32779137 PMC7572954

[25] Char DS, Abràmoff MD, Feudtner C. Identifying ethical considerations for machine learning healthcare applications. Am J Bioeth. 2020;20(11):7–17. doi: 10.1080/15265161.2020.1819469 PMC7737650 33103967

[26] Vollmer S, Mateen BA, Bohner G, Machine learning and artificial intelligence research for patient benefit: 20 critical questions on transparency, replicability, ethics, and effectiveness. BMJ. 2020;368:l6927. doi: 10.1136/bmj.l6927 32198138 PMC11515850

[27] Badal K, Lee CM, Esserman LJ. Guiding principles for the responsible development of artificial intelligence tools for healthcare. Commun Med. 2023;3(1):47. doi: 10.1038/s43856-023-00279-9 37005467 PMC10066953

[28] Gunning D, Aha DW. DARPA’s explainable artificial intelligence program. AI Mag. 2019;40(2):44–58. doi: 10.1609/aimag.v40i2.2850

[29] US Department of Health and Human Services. Summary of the HIPAA Privacy Rule. 2022. https://www.hhs.gov/hipaa/for-professionals/privacy/laws-regulations/index.html

