# Supplementary material for: Artificial intelligence as a tool for cognitive impairment screening: Patient perspectives about benefits and limitations
Source: Alzheimers Dement Behav Socioecon Aging. Author manuscript; Available in PMC 2026 Aug 27. (PMC13508175; doi:10.1002/bsa3.70005)
Supplement: Supplementary file 1 [file NIHMS2201354-supplement-Artificial_intelligence_as_a_tool_for_cognitive_impairment_screening__Patient_perspectives_about_benefits_and_limitations_Supplemental_A__Interview_Guide.pdf]

Supplemental A: Interview Guide

Cognitive Impairment

Today we're talking about dementia and cognitive impairment.

Are you familiar with the term "dementia" or "Alzheimer's disease"?

- Can you tell me what you know about dementia?
- Can you tell me what you know about Alzheimer's disease?
- Have you known someone with dementia or Alzheimer's disease?
  - Tell me about people you know with dementia – how does dementia affect them?

Have you heard of the term "cognitive impairment"? What does it mean?

[Explain]

- When a person has cognitive impairment (CI) it means some brain functions aren't working normally
- For example, they may have trouble remembering, have difficulty concentrating, or struggle to do simple math
- In mild CI, the problems may be hardly noticeable, and usually, people only complain about forgetting things;
- But in dementia, which is severe CI, other people notice the problems; people with dementia always need help doing even simple things, like taking medications or bathing themselves
- Current treatments available to clinicians do not cure dementia.

Screening

In your own words, how would you describe a health screening?

[Explain if needed]

- A health screening is a test that is used to see if a person has a health problem. There are many types of screening tests. For example, your primary clinician conducts basic tests to evaluate your eyesight. Or, a colonoscopy is a screening test that uses a special camera to look for colon cancer, and there's a blood test to screen for high cholesterol.

There are also screening tests for CI. These are usually questions that a doctor asks a patient to test memory, concentration, ability to solve problems and other cognitive skills. A positive screening test result may lead to additional testing, like blood tests or a brain scan, to help determine a diagnosis.

Computer Technology in the Clinic

In this section, we are going to talk about AI or artificial intelligence. When I say "artificial intelligence" what do you think of?

[Explain:] *AI are computer programs that use data to make predictions or identify things. For example, when you use the Internet do you find that advertisements pop up that seem to be designed just for you. A computer program using AI figures out what you like and then sends you advertisements that match your interests.*

Before we move on do you have any questions about AI?

In these next few questions, we will talk about how clinicians might use AI in medical practice, for example diagnosing an illness.

- *Research evidence shows that cognitive impairment and dementia, including Alzheimer's disease, is underdiagnosed or misdiagnosed. This may be, in part, because primary care doctors lack the time and specialty training on dementia and cognitive impairment.*
- *One solution may be to use a type of AI that would automatically screen patients for cognitive impairment.*
- *Doctors aren't yet using AI yet to screen for CI*
- *If they were, this is how it might work: a computer would use the data in your medical record to determine if you have CI and then send a message to your PCP with the answer.*
- *Your PCP could tell you the result, and make decisions about other tests to do, as well as treatments and counseling or support to provide.*

For you, what would be benefits of a cognitive screening using AI?

For you, what harms would you be concerned about?

The AI technology would operate automatically – giving the doctor information about your cognitive function as soon as they open your medical record. A patient might not be aware that the computer was evaluating their cognition.

If you found out your doctor had used AI to automatically screen you for cognitive impairment, what would your reaction be?

Consent procedures

Would you want to know that AI was being used to screen you for cognitive impairment?

There are different options for getting your permission regarding the screening using AI. I will describe them, then you tell me what you think of each:

1.The first option would be: The doctor would automatically screen you for CI by using AI to evaluate your medical records. They would only notify you IF the screening found something that raised concern. Then, at that point, the doctor would ask you if you would like to know the results. And then you would have the option to learn or not learn that information.

2.The second option would be: You would be notified BEFORE your appointment that you would be screened for CI by using AI to evaluate your medical records. You would then be given the option to “opt out” or decline the screening prior to your appointment.

So, the difference between the two options is, in the first option you would not give permission before the technology (AI) was used to screen you for CI. In the second option, you would be told about the technology and could opt out from the screening.

Tell me your thoughts about these two options?

PROMPT: Tell me your thoughts about getting patient’s consent to do automated CI screening.

[Prompt:]

- Should the doctor ask your permission to use your medical record data to see if you have CI?
- Should the doctor ask your permission to give you the results of CI screening?

(Transparency/Consent):

What would you want the doctor to explain to you about the automatic screening?

Automated Screening Using Voice Analysis

- Another way of automatically screening for CI would be to use a recording of your voice
- A computer program would analyze your voice, just like it might analyze data from your medical record, to determine if you have CI
- Here’s how this might work: during a PCP visit, a microphone connected to your doctor’s computer would record your conversation. It would then analyze the recording and send a message to the doctor saying whether or not you have CI.

Tell me your thoughts about screening for CI this way?

(Benefits/harm evaluation):

What are some <u>benefits</u> of screening for CI using voice recordings?

What are some <u>harms</u> of screening for CI using voice recordings?

When we discussed consent for automated screening, you said XYZ. Would you feel the same about consent for this type of screening?

Wrap up.

End of the interview
