# Supplementary material for: Artificial intelligence as a tool for cognitive impairment screening: Patient perspectives about benefits and limitations
Source: Alzheimers Dement Behav Socioecon Aging. Author manuscript; Available in PMC 2026 Aug 27. (PMC13508175; doi:10.1002/bsa3.70005)

Supplemental B: Infographic for explaining consent scenarios

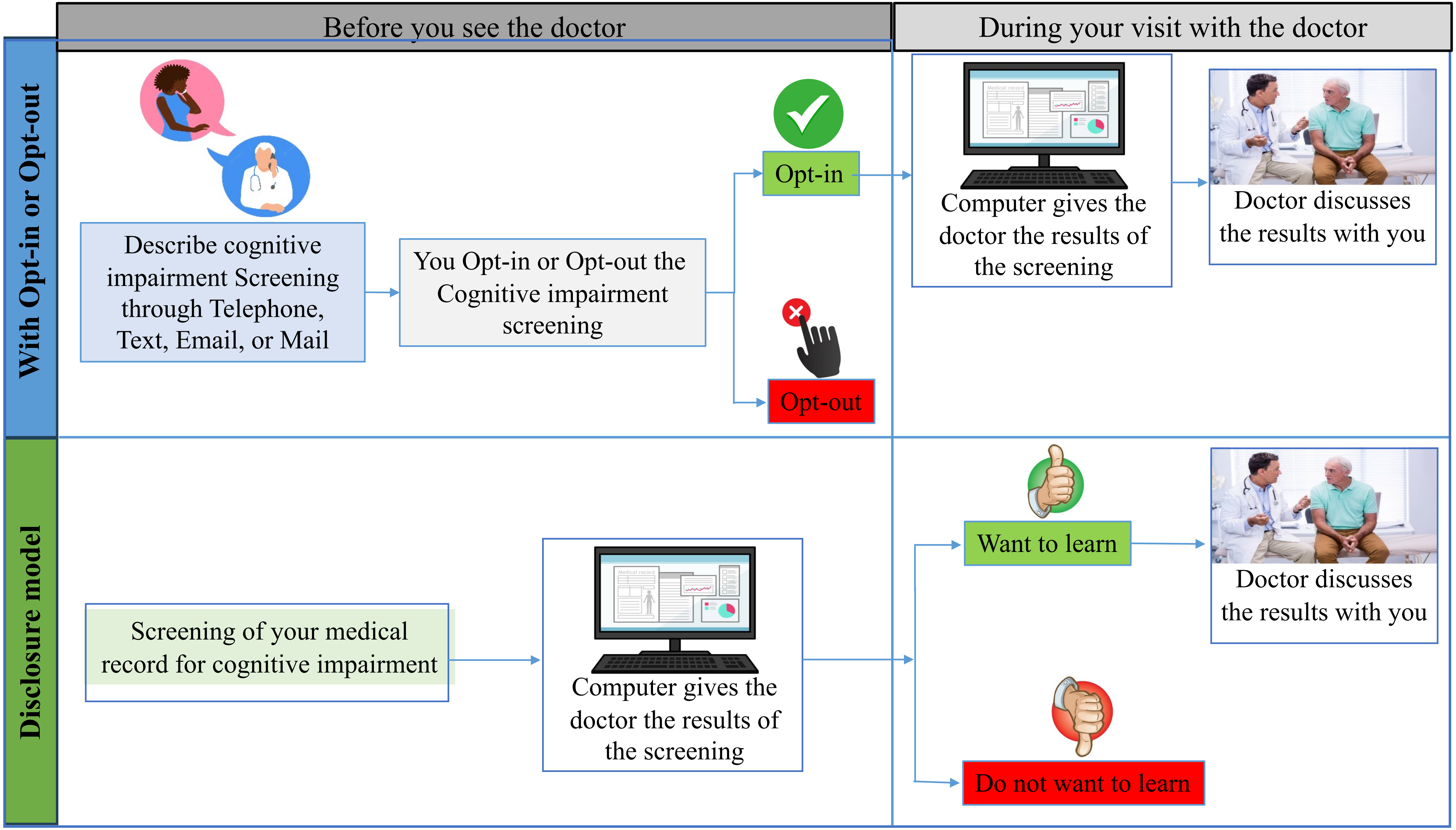

Supplement: Supplementary file 2 [file NIHMS2201354-supplement-Artificial_intelligence_as_a_tool_for_cognitive_impairment_screening__Patient_perspectives_about_benefits_and_limitations__Supplemental_B_Consent_Infographic.pdf]
